# Fluoride-Treated Nano-HZSM-5 Zeolite as a Highly Stable Catalyst for the Conversion of Bioethanol to Propylene

**DOI:** 10.3390/nano14191558

**Published:** 2024-09-26

**Authors:** Jian Zhou, Ni Zhang, Tao Meng, Qiangsheng Guo, Zhaoteng Xue, Dongsen Mao

**Affiliations:** School of Chemical and Environmental Engineering, Shanghai Institute of Technology, Shanghai 201418, China; 216061106@mail.sit.edu.cn (J.Z.); 146061314@mail.sit.edu.cn (N.Z.); mengtao@sit.edu.cn (T.M.); guoqsh@sit.edu.cn (Q.G.)

**Keywords:** fluoride modification, NH_4_F-HF, ZSM-5 zeolite, bioethanol to propylene

## Abstract

Fluoride treatment of ZSM-5 zeolite can effectively adjust surface acidity and generate a secondary pore structure. In this study, a series of modified nano-HZSM-5 zeolites were prepared by NH_4_F-HF mixed solution treatment and applied to the selective conversion of bioethanol to propylene at 500 °C, atmospheric pressure, and a WHSV of 10 h^−1^. The results showed that NH_4_F-HF modification weakened the surface acidity of nano-HZSM-5 zeolites, thus inhibiting coke formation. Additionally, the mesopores in the nano-HZSM-5 zeolites increased after NH_4_F-HF treatment, thereby enhancing the mass transfer rate and improving the coke-resistance ability. The NH_4_F-HF mixed solution modification significantly improved the stability of nano-HZSM-5 zeolites in catalyzing bioethanol to propylene and greatly extended the working life of nano-HZSM-5 zeolites. It can be seen from the characterization of the deactivated catalysts that coke deposition and weakening of acidity may be the key factors for catalyst deactivation.

## 1. Introduction

Nowadays, propylene is one of the largest petrochemical products after ethylene. Meanwhile, it is also one of the basic raw materials of the three major synthetic materials which can be used to produce polypropylene, acrylonitrile, acetone, etc. At present, the traditional propylene production processes mainly include steam cracking, catalytic cracking, methanol to olefins, propane dehydrogenation, and olefin metathesis. However, due to the long-term tight supply-and-demand situation of propylene, coupled with the maturity of bioethanol development and utilization technologies in recent years, the ethanol-to-propylene (ETP) process has been rapidly developed as a green and environmentally friendly propylene production technology. This process uses renewable bioethanol, which avoids the environmental pollution and climate change associated with traditional fossil fuels [1,2,3,4,5].

Catalysts for ethanol-to-propylene conversion are generally categorized into two types: metal oxide catalysts [6,7,8] and zeolite catalysts [9,10,11,12]. Among them, ZSM-5 zeolite has shown superior performance in the process of catalytic conversion of ethanol to propylene due to its remarkable acidity, unique framework, and pore structure. Specifically, ZSM-5 zeolite has exhibited excellent thermal stability, good anti-coking ability, and exceptional shape-selective catalytic properties [13]. These characteristics not only make it the focus of this research field, but have also led to its widespread application in various reaction systems [14,15,16,17,18], laying the foundation for further studies on zeolite modification. By surface modification or doping with other metals, the acid center and pore structure of ZSM-5 zeolite can be effectively adjusted, thereby mitigating the disadvantages of low propylene selectivity and rapid deactivation of conventional ZSM-5 zeolite in ETP reaction due to the diffusion limitation of micropores and strong acidity [7,19,20,21].

Fluoride treatment of ZSM–5 zeolite [22,23,24,25,26,27] is an effective method to tune its surface acid properties. Previously, our team [28] prepared a series of HF-modified nano-ZSM-5 zeolite catalysts by post-treatment with different concentrations of hydrofluoric acid (HF) solution for selective conversion of bioethanol to propylene. The results showed that the acid strength and the number of acid sites decreased significantly after the zeolite was treated with HF solution. The propylene selectivity and working life of nano-ZSM-5 zeolite can be significantly improved and prolonged by HF solution modification with a suitable concentration (mass fraction, 6%). For ZSM-5 zeolite, the weakening of its acidity can inhibit the formation of coke during the reaction and slow down the coke deposition rate, thus prolonging its working life. However, the catalytic performance of zeolite is still limited by its pore structure. If the reaction product fails to diffuse to the external surface of the catalyst in time, it will lead to the formation of coke precursors due to the secondary reaction of the product [29,30,31]. The formation of coke precursors will not only decrease the propylene selectivity, but also cause a rapid decline in the stability of the catalyst. Therefore, it is essential to further optimize the pore channel of the nano-ZSM-5 zeolite after treatment by HF.

Previous studies [22,27,32,33,34] have shown that ammonium fluoride (NH_4_F) treatment had an obvious pore expansion effect on the zeolite, leading to a significant increase in the external specific surface area, mesopore volume, and average pore diameter. Qin et al. [35] found that a dilute HF solution (<1 mol/L) could preferentially extract the framework aluminum in the zeolite, resulting in a significant increase in the Si/Al ratio and a substantial decrease in the number of Brønsted acid sites. By introducing NH_4_F buffer solution into the HF solution and properly adjusting the concentration of both (HF and NH_4_F solutions), the silicon and aluminum framework in the zeolite could be extracted simultaneously, thus keeping the Si/Al ratio similar to the parent, so the acidity of the catalyst could be retained. At the same time, after treatment with the NH_4_F-HF mixed solution, a secondary pore structure could also be generated in the zeolite, which facilitated the diffusion of the product, thereby increasing the conversion of m–xylene. In addition, Qin et al. [35] also discovered that the NH_4_F-HF mixed solution could preferentially dissolve the defect region in the ZSM-5 zeolite, resulting in a significant reduction in defects (silanols), which was beneficial to the catalytic reaction deactivated by coke deposition. A similar finding was also obtained by Luo et al. [36]: after the fluorination treatment (NH_4_F-HF), the terminal silanol groups in the ZSM-5 zeolite decreased after reacting with the F species. Meng et al. [37] found that, compared to HF solution treatment, NH_4_F-HF mixed solution treatment preserved the initial chemical composition of the ZSM-5 zeolite. The retention of acidity and the formation of mesopores significantly extended the catalytic life of ZSM-5 zeolite in the methanol-to-gasoline reaction.

To the best of our knowledge, studies on the use of NH_4_F-HF mixed solution to treat HZSM-5 zeolite for the catalytic conversion of ethanol to propylene remain unreported. Moreover, most previous studies on the selective conversion of ethanol to propylene using the HZSM-5 zeolite had primarily utilized pure ethanol as the feedstock. However, obtaining pure ethanol or high–purity ethanol (>92%) is more costly. Therefore, the use of bioethanol (dilute ethanol containing water) directly would be more cost-effective [38,39]. This study aims to modify nano-HZSM-5 zeolite with an appropriately proportioned NH_4_F-HF mixed solution to achieve superior catalytic performance in the ETP process. Additionally, the structure–activity relationships between the structure and surface acid properties of the zeolite and its catalytic reaction performance are thoroughly discussed.

## 2. Experimental

### 2.1. Catalyst Preparation

First, HF (Shanghai Titan Scientific Co., Ltd., Shanghai, China) aqueous solutions with mass fractions of 2%, 6%, and 8% were prepared (the corresponding mass is 10.1 g, 10.3 g, and 10.4 g, respectively), and then 2 g of solid NH_4_F (Shanghai Titan Scientific Co., Ltd., Shanghai, China) particles were added to each solution and stirred until completely dissolved to obtain NH_4_F-HF mixed solutions of varying ratios. Subsequently, the mixed solutions were added, respectively, to 5 g of HZSM-5 zeolite (produced by Zibo Tengjin Energy Saving Technology Co., Ltd., Zibo, Shandong, China, with a Si/Al molar ratio of 16.9 and an average crystal size of 80 nm) using the isovolume impregnation method. The mixture was placed at room temperature for 3 h, dried at 110 °C for 12 h, and then calcined at 500 °C for 4 h to obtain the desired samples. The samples were named HZ-2NH, HZ-6NH, and HZ-8NH according to the HF concentration in the NH_4_F-HF mixed solution, while the unmodified sample was named HZ.

### 2.2. Catalyst Characterization

The crystal structure of the catalysts was analyzed using an X’Pert PRO PW3040/60 X-ray diffractometer (Cu Kα radiation, 40 kV, 40 mA, 2θ = 5–50°, 4°/min) from the PANalytical, Almelo, Netherlands. The relative crystallinity of the ZSM-5 zeolite samples was calculated based on the sum of the peak areas of the diffraction peaks in the 2θ = 22–25° range from the XRD patterns [40]. Elemental analysis of the samples was conducted using a Vista-MPX inductively coupled plasma optical emission spectrometer (ICP-OES) from the Varian, Palo Alto, CA, USA. The specific surface area and pore parameters of the samples were measured using an ASAP 2020 HD88 adsorption analyzer from the Micromeritics, Norcross, GA, USA. Prior to measurement, the samples were degassed at 200 °C for 13 h and subsequently underwent a full micro–mesopore analysis under liquid nitrogen isothermal conditions (−196 °C). Total surface area was obtained by the Brunauer-Emmett-Teller (BET) equation, the total pore volume was calculated by the single point method, and the micropore area and volume were calculated according to the t-plot method. The external area was acquired by subtracting the micropore area from the total BET surface area. The mesopore volume was obtained by subtracting the micropore volume from the total pore volume. The average pore diameter was measured by the BJH method. The surface acidity of the samples was analyzed using a temperature-programmed desorption of ammonia (NH_3_-TPD) apparatus, with the signals collected by a GC9750 gas chromatograph equipped with a thermal conductivity detector (TCD) from the Zhejiang Fuli Analytical Instrument Co., Ltd., Wenling, Zhejiang, China. First, the samples (0.10 g) were pretreated at 500 °C for 60 min, then cooled to room temperature and saturated with 10% NH_3_/N_2_ (mol/mol). Afterward, the samples were purged with high-purity N_2_ at 100 °C for 60 min. Finally, the temperature was increased from 100 °C to 550 °C at a rate of 10 °C min^−1^ while data were collected. The coke deposition content of the deactivated samples was measured using a STA 449 F3 thermogravimetric (TG) analyzer from NETZSCH, Selb, Bavaria, Germany. The deactivated samples were heated from 30 °C to 800 °C at a rate of 10 °C/min under an air atmosphere. The average coke deposition rate [41] was calculated from the content of coke deposition (weight loss between 400 °C and 700 °C), the mass of the catalyst, and the catalytic reaction time.

### 2.3. Catalytic Conversion of Bioethanol

The catalytic performance was evaluated using a continuous-flow micro fixed-bed reactor (with an inner diameter of 6 mm). First, 0.3 g of catalyst was pretreated at 500 °C for 60 min in a nitrogen atmosphere. Subsequently, the ethanol aqueous solution (90 vol%) was introduced into the catalytic bed using a small-flow feed pump, controlling the weight hourly space velocity (WHSV) of ethanol at 10 h^−1^. The temperature of 500 °C and WHSV of ethanol at 10 h^−1^ were selected based on our previous study [28]. The products were analyzed by an Agilent 6820 gas chromatograph equipped with a flame ionization detector (FID, temperature: 250 °C) and an HP-Plot-Q capillary column (19091P-QO4, column temperature: 250 °C) with highly pure nitrogen as carrier gas. The line between the reactor outlet and the gas chromatograph inlet was kept warm with a heating tape (maintained at 180 °C) to prevent product condensation.

The calculations for ethanol conversion (*x*, %) and product selectivity (*s_i_*, %) are shown in Equations (1) and (2), respectively.

(1) $$x=\frac{n_{EtOH\left(in\right)}-n_{EtOH\left(out\right)}}{n_{EtOH\left(in\right)}}\times 100\%$$

(2) $$s_{i}=\frac{v_{i}c_{i}}{\sum v_{i}c_{i}}\times 100\%$$

where *n*_*EtOH*(*in*)_ is the moles of ethanol at the inlet (mol); *n*_*EtOH*(*out*)_ is the moles of ethanol at the outlet (mol); *v_i_* is the number of carbon atoms in the *i*th product; and *c_i_* is the molar concentration of the *i*th product (mol/L).

## 3. Results and Discussion

### 3.1. Characterization of the Catalysts

**Figure 1:**
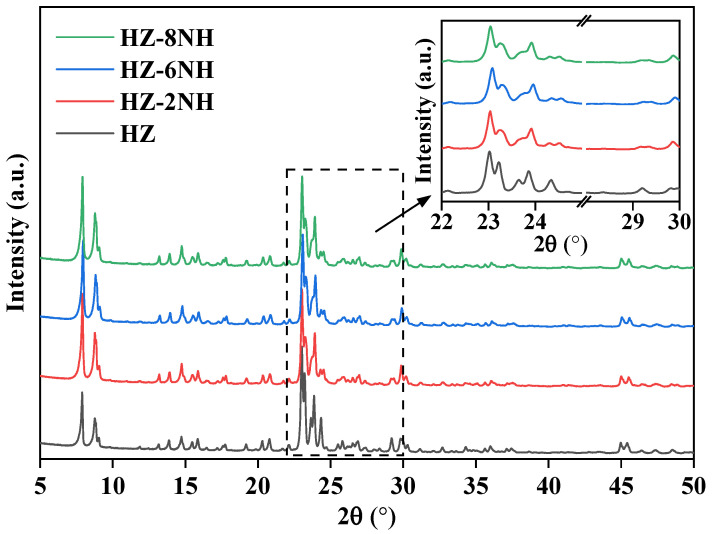
XRD patterns of different samples.

As shown by the XRD patterns of different samples in Figure 1, the NH_4_F-HF-modified samples retained their original MFI configuration compared to the unmodified HZ. However, the diffraction peak intensity was significantly weakened, and the relative crystallinity decreased from 100% in HZ to ~76% (Table 1). This reduction was likely due to the leaching of framework silicon and aluminum from the zeolites by the NH_4_F-HF mixed solution [35], leading to a certain degree of structural damage to the catalysts. Additionally, the diffraction peaks at 24.4° and 29.2° for HZ appeared as single peaks, while after modification, these peaks split into doublets. This indicated that the crystal symmetry of part of the catalysts had transitioned from orthorhombic to monoclinic symmetry [42].

Unlike modification with HF solution, modification with the NH_4_F-HF mixed solution did not significantly alter the Si/Al molar ratio of the HZSM-5 zeolites (Table 1), which was consistent with the findings of Qin et al. [35]. It is well known that HF is a weak acid and in an aqueous solution exists in the following ionization equilibrium:

HF ⇋ H^+^ + F^−^ (3)

HF + F^−^ ⇋ HF_2_^−^ (4)

The introduction of NH_4_F into the HF aqueous solution increased the concentration of F^−^ in the mixed solution, causing the ionization equilibrium (3) shift to the left and the ionization equilibrium (4) shift to the right, which led to an increase in the concentrations of HF and HF_2_^−^ in the mixed solution. As we knew, both HF and HF_2_^−^ were capable of removing framework silicon from the zeolite, and the higher their concentrations, the faster the rate of desilication [43,44]. Therefore, compared to the dilute HF solution, the NH_4_F-HF mixed solution increased the desilication selectivity from the zeolite, rather than preferentially dealuminating. In other words, the NH_4_F-HF mixed solution removed framework silicon and framework aluminum from the zeolite simultaneously, which resulted in the Si/Al molar ratio of the modified catalysts remaining largely unchanged compared to HZ.

**Figure 2:**
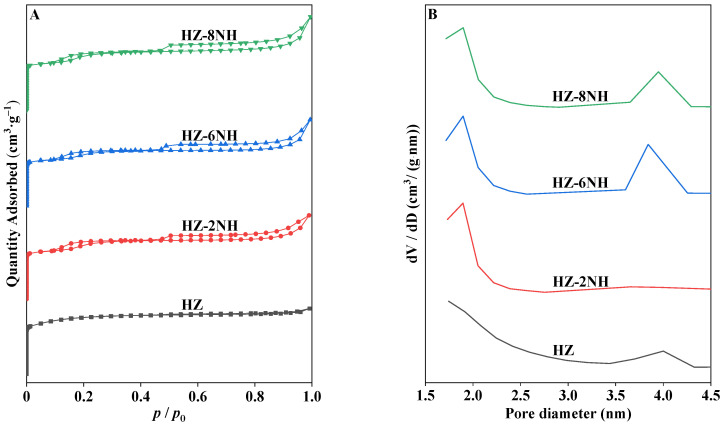
N_2_ adsorption–desorption curves (**A**) and pore diameter distributions (**B**) of different samples.

The NH_4_F-HF modification significantly altered the pore structure of the zeolite. As shown in Table 1, after modification, the micropore specific surface area of the zeolite was significantly reduced, while the external specific surface area increased markedly. Simultaneously, the micropore volume decreased, the mesopore volume increased, the total pore volume increased from 0.19 cm^3^ g^−1^ to 0.27 cm^3^ g^−1^, and the average mesoporous diameter expanded from 2.30 nm to 3.24 nm. These phenomena indicate that some micropores in the modified samples were converted into mesopores [26], a conclusion that could also be drawn from the nitrogen adsorption–desorption curves and pore diameter distributions (Figure 2). As shown in Figure 2, HZ displayed the characteristics of Type I adsorption isotherms (IUPAC). At very low *p*/*p*_0_, the adsorption quantity increased sharply due to the enhanced interaction between the zeolite and nitrogen in the narrow micropores, leading to micropore-filling at extremely low relative pressure. As *p*/*p*_0_ increased, the adsorption quantity gradually stabilized, exhibiting an almost horizontal trend [45]. Two hysteresis loops were observed in the modified samples HZ-2NH~HZ-8NH. The hysteresis loop located in the *p*/*p*_0_ range of 0.1~0.3 resulted from the phase transition of the adsorbed nitrogen from a disordered state to a crystalline state [46]. The hysteresis loop in the *p*/*p*_0_ range of 0.5~1.0 resulted from capillary condensation in the mesopores [29]. Additionally, as the HF concentration in the NH_4_F-HF mixed solution increased, the integral area of the hysteresis loop in the 0.5~1.0 range gradually expanded, indicating that more mesopores were formed in the modified samples. This was the result of the synergistic effect of NH_4_F [27] and HF [28]. Meanwhile, in the pore diameter distribution (Figure 2B), the peak at 4.0 nm was more pronounced in HZ-6NH, further corroborating the creation of new mesopores.

**Figure 3:**
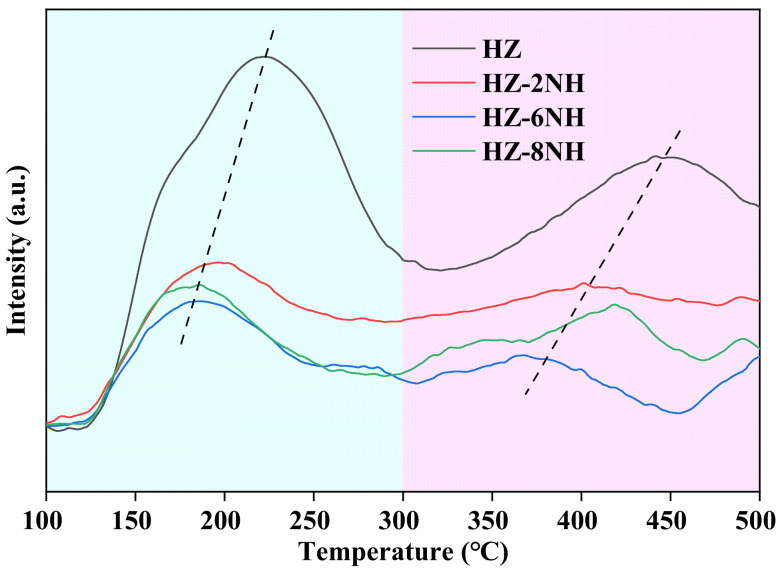
NH_3_-TPD curves of different samples.

NH_3_-TPD was performed to reveal the effect of NH_4_F-HF modification on the surface acidity of the catalysts. As shown in Figure 3, all samples showed a low-temperature desorption peak and a high-temperature desorption peak before and after 300 °C, corresponding to the weak acid site and strong acid site of the catalysts, respectively. After treatment with the NH_4_F-HF mixed solution, the peak areas of both the low-temperature and high-temperature desorption peaks of the catalysts were significantly decreased (Table S1 in Supporting Information), indicating a substantial reduction in the number of weak and strong acid sites. Meanwhile, the two desorption peaks of the modified samples shifted toward lower temperatures, indicating the acid strength of both the weak and strong acid sites was weakened. This was due to the conversion of some framework aluminum in the zeolite into non-framework aluminum by the NH_4_F-HF modification, leading to a weakening in the surface acidity of the catalyst [36]. Moreover, compared to HZ-6NH, HZ-8NH exhibited an increase in both acid amount and acid strength, which may have been due to the incorporation of highly electronegative fluoride ions into the framework of HZ-8NH. This incorporation may have polarized some structures, thereby enhancing the surface acidity [35].

### 3.2. Catalytic Performance

The effect of NH_4_F-HF modification on nano-HZSM-5 zeolite in the catalytic conversion of bioethanol to propylene was evaluated under reaction conditions of atmospheric pressure, 500 °C, and an ethanol WHSV of 10 h^−1^. It has been well demonstrated that the addition of a small amount of water can mitigate the HZSM-5 deactivation caused by coke formation. However, the addition of a large amount of water can result in irreversible deactivation caused by partial dealumination of HZSM-5 [19,47,48]. On the other hand, it is well known that the purification of ethanol from 90% to higher purity is particularly energy-consuming. Considering these aspects, in our previous work we used the 90% ethanol as bio-ethanol in the ETP reaction over H-ZSM-5. For making a fair comparison of the effect of various post-treatments on the performance of HZSM-5, the same feed composition as in our previous studies (90%) was therefore used in this work. Throughout the reaction, the ethanol conversion rate consistently remained at 100%.

To facilitate the study of the distribution of reaction products, Table 2 lists the distribution of various products in the initial reaction stage, including ethylene, propylene, butylene, C_1_–C_4_ alkanes, and aromatics. As shown in Table 2, the initial selectivity of different catalysts for the same product varies. After treatment with the NH_4_F-HF mixed solution, the selectivity for light olefins (ethylene, propylene, and butylene) was significantly increased, while the selectivity for C_1_–C_4_ alkanes and aromatics was significantly decreased. Based on the reaction mechanism of ethanol-to-propylene conversion over ZSM-5 zeolite [10,11,12,29], ethylene was initially produced from ethanol and was easily formed in a weakly acidic environment. Subsequently, ethylene oligomerized to form olefins, which either cracked into propylene and butylene or underwent hydrogen transfer or aromatization to form alkanes, aromatics, and other higher hydrocarbons. The hydrogen transfer and aromatization processes required a strongly acidic environment to proceed [49,50,51]. Therefore, HZ with stronger acidity showed higher selectivity for C_1_–C_4_ alkanes and aromatics, while its selectivity for light olefins (ethylene, propylene, and butylene) was lower. The acidity of the modified samples was greatly weakened after treatment with the NH_4_F–HF mixed solution (Figure 3). As a result, the selectivity for light olefins (ethylene, propylene, and butylene) increased as the catalyst acidity weakened, while the selectivity for C_1_-C_4_ alkanes and aromatics decreased. As shown in Figure 3, the acidity of HZ-8NH was somewhat stronger than that of HZ-6NH, but its ethylene selectivity was higher than that of HZ-6NH (the relative increase of 4.1%, which is higher than the relative error of selectivity analysis of less than 2%). This could be attributed to the larger external specific surface area and mesopore volume of HZ-8NH (Table 1), indicating that both the pore structure and acidity of the catalyst played roles in determining product selectivity [52,53].

**Figure 4:**
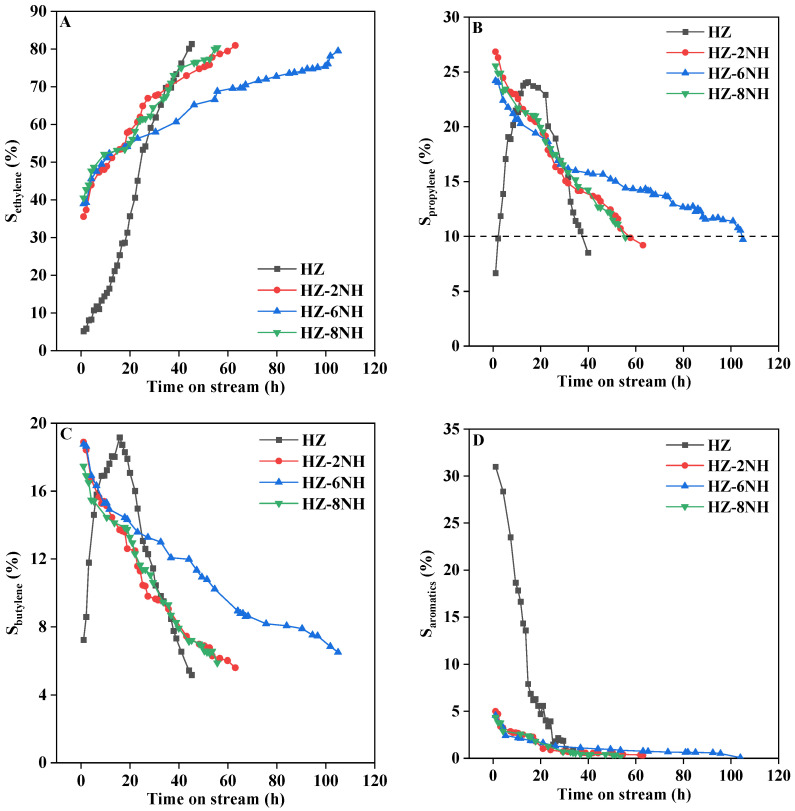
Variation of C_2_–C_4_ olefin and aromatic selectivities with time on stream for different samples. (**A**) Ethylene selectivity varies with time on stream. (**B**) Propylene selectivity varies with time on stream. (**C**) Butylene selectivity varies with time on stream. (**D**) Aromatics selectivity varies with time on stream.

Figure 4 illustrates the variation in C_2_–C_4_ olefin and aromatic selectivity over different samples as the reaction progressed. As shown in Figure 4A, the selectivity for ethylene gradually increased as the reaction progressed for all samples, while the selectivity for aromatics exhibited the opposite trend (Figure 4D); as the reaction proceeded, the selectivity for aromatics steadily declined. Relevant studies [41,54,55] indicated that ZSM-5 zeolites with weaker surface acidity facilitated ethanol conversion to ethylene, while those with stronger surface acidity tended to produce higher-carbon hydrocarbons such as aromatics. During the ethanol-to-propylene conversion process, the strong acid sites of the ZSM-5 zeolite were first deactivated by coke formation, which consumed some of the strong acid sites. As a result, the selectivity for aromatics diminished as the acidity weakened. The reduction in the number of strong acid sites led to a weaker acidic environment on the catalyst surface, which promoted ethylene formation. Additionally, the reduced number of strong acid sites prevented the already-formed ethylene from undergoing hydrogen transfer or aromatization to form other products, causing ethylene selectivity to gradually increase as the reaction progressed. This indicated that the deactivation of the strong acid sites on the ZSM-5 zeolite catalyst played a key role in driving the selectivity trends of ethylene and aromatics [29]. As shown in Figure 4B, the propylene selectivity of HZ initially increased and then decreased as the reaction progressed, reaching its maximum value (24.1%) at 14.7 h. However, the modified samples (HZ-2NH, HZ-6NH, and HZ-8NH) showed a different behavior; they exhibited the highest propylene selectivity at the beginning of the reaction, which then gradually decreased. This was because the modified samples had fewer strong acid sites that favored aromatic formation (Figure 3), which led to lower aromatic selectivity (Figure 4D). The proportion of strong acid sites, which were conducive to propylene formation, was at its highest at the beginning of the reaction and then gradually decreased as the reaction progressed. Therefore, propylene selectivity peaked at the start of the reaction and then gradually decreased. Additionally, the trend in butylene selectivity (Figure 4C) was similar to that of propylene, which could be attributed to the fact that their formation reactions occurred in parallel [27,41,51].

As shown in Figure 4B, the working life (measured by the reaction time during which propylene selectivity remained ≥ 10%) of different samples differed. For HZ, its working time proved to be relatively short (36.8 h), which could be attributed to its strong surface acidity (Figure 3). As previously mentioned, a stronger acidic environment tended to promote the formation of higher-carbon hydrocarbons such as aromatics, and coke was generated through the polycondensation of aromatics [56]. Therefore, the rapid deactivation of the catalyst was caused by the heavy coke deposition on its surface, leading to a shorter working life. The modified samples, however, exhibited longer working life than HZ, which could be attributed not only to the weakening in acidity that inhibited aromatic formation (Figure 4D), but also to the increase in mesopores (Table 1). The increased mesopores made it easier for products like propylene to diffuse out of the pores, reducing secondary reactions of higher-carbon hydrocarbons like aromatics. Meanwhile, it was known that mesopores were the primary sites for coke deposition [29,57]. Therefore, the significant increase in the external specific surface area of the modified samples (Table 1) improved their resistance to coke, allowing them to accommodate more coke and thereby prolonging the catalytic lifespan. This demonstrated that both the acidity and pore structure of the ZSM-5 zeolite catalyst played significant roles in affecting the stability of the catalytic process [52,53]. HZ-2NH and HZ-8NH showed a similar working life (57.8 h and 55.7 h, respectively), while HZ-6NH had the longest working life, reaching 105 h. This was approximately three times the catalytic lifespan of HZ and also longer than the working life (95 h) of the sample treated with HF solution (ZSM-5-6HF) from our previous work [28]. Therefore, HZ-6NH exhibited the best catalytic stability among all samples.

### 3.3. Characterization of Deactivated Catalysts

**Figure 5:**
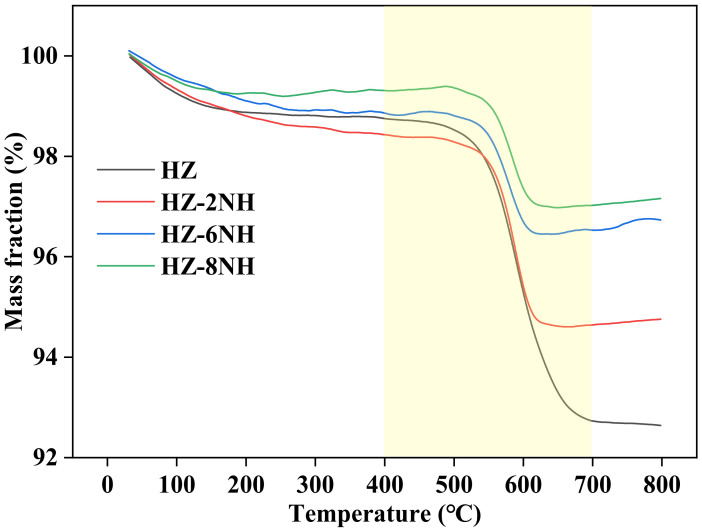
TG curves of different spent samples.

After the reaction, the color of the catalysts changed from white to black, indicating that coke formation was likely one of the main causes of catalyst deactivation [27]. Therefore, thermogravimetric analysis was performed to study the deactivated catalysts. Figure 5 shows the TG curves of the spent catalysts (the corresponding DTG curves are shown in Figure S1), where a noticeable weight loss was observed in the range of 400~700 °C, which was attributed to the combustion of coke on the deactivated catalysts. Based on the weight loss, the relative coke contents of HZ, HZ–2NH, HZ–6NH, and HZ–8NH were calculated as 6.6%, 3.7%, 2.4%, and 2.3%, respectively, and using the reaction time of each sample (44, 63, 105, and 56 h), the average coke deposition rates were calculated as 1.4 × 10^−3^, 5.9 × 10^−4^, 2.3 × 10^−4^, and 4.1 × 10^−4^ g/(g_cat_·h), respectively. The calculated results indicated that the average coke deposition rates of the modified samples had significantly decreased compared to that of HZ. This decrease could be attributed to the formation of new mesopores in the modified samples, which enhanced mass transfer rates and reduced the formation of coke precursors. Additionally, the weakening of acidity in the modified samples reduced the likelihood of forming coke precursors—aromatics—thereby inhibiting coke formation.

**Figure 6:**
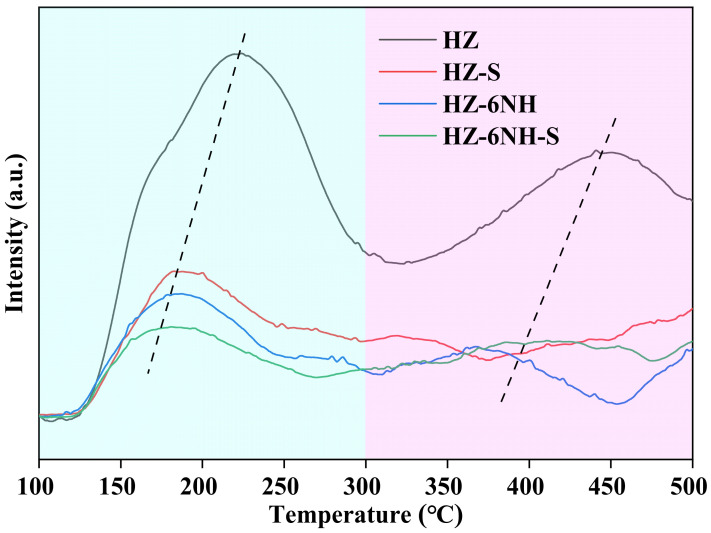
NH_3_-TPD curves of fresh and spent samples.

NH_3_-TPD was conducted on deactivated HZ and HZ-6NH (named HZ-S and HZ-6NH-S, respectively, with “S” indicating deactivation) to investigate the changes in acidity before and after the reaction. As shown in Figure 6, compared to the fresh catalysts (HZ and HZ-6NH), both the low-temperature and high-temperature desorption peaks of HZ-S and HZ-6NH-S shifted toward a lower temperature, indicating that the acid strength of both the weak and strong acid sites had weakened. Additionally, the peak area of the low-temperature desorption peaks of the deactivated samples significantly decreased, and the high-temperature desorption peaks almost disappeared, indicating a substantial reduction in the number of weak acid sites and the near disappearance of strong acid sites. Based on the above results, it could be inferred that coke covered the acid sites of the catalyst, leading to a decline in its catalytic performance [29,41].

**Figure 7:**
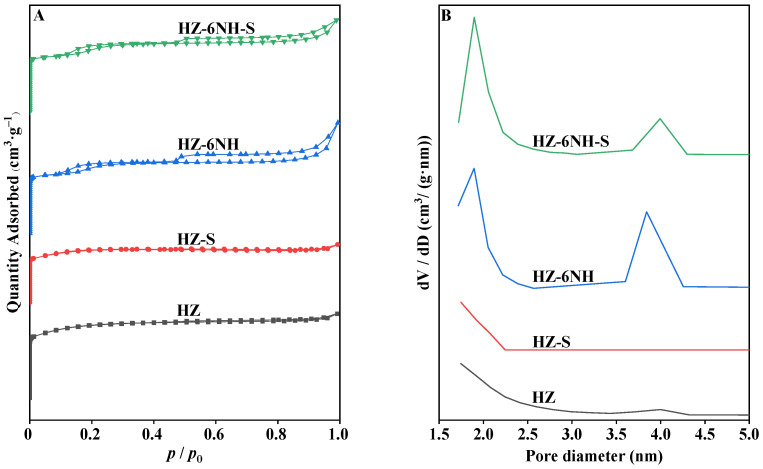
N_2_ adsorption–desorption curves (**A**) and pore diameter distributions (**B**) of fresh and spent samples.

N_2_ adsorption–desorption analysis was used to analyze the changes in pore structure before and after the reaction. As shown in Figure 7A, the hysteresis loop area in the *p*/*p*_0_ range of 0.5~1.0 for the deactivated samples significantly decreased, and the peak at 4.0 nm in the pore diameter distributions (Figure 7B) and the average pore diameter (Table 3) both decreased. These phenomena indicate that most of the coke was deposited in the mesopores of the catalyst [29,57]. As shown in Table 3, compared to HZ, the specific surface area and pore volume of HZ-S significantly decreased, which likely contributed to the deactivation of the catalyst. For HZ-6NH and HZ-6NH-S, however, their specific surface area and pore volume were similar, so the deactivation was likely explained by the weakening of acidity. This further demonstrated that both the acidity and pore structure of the catalysts played critical roles in affecting their stability [52,53].

The deactivated catalysts were regenerated by calcination in air flow at 600 °C for 5 h. The performance of the regenerated catalysts was evaluated, and the results are consistent with those of the fresh catalysts.

## 4. Comparison of NH_4_F-HF Modification and HF Modification

In this study, nano-HZSM-5 zeolites were modified with an appropriately proportioned NH_4_F-HF mixed solution, to enhance their catalytic performance in the ethanol-to-propylene process. It is well known that the propene selectivity and catalyst stability of HZSM-5 catalysts for the ETP reaction are greatly dependent on the reaction conditions such as temperature and ethanol WHSV, and feed composition. Additionally, most of the former studies used the mixture of vaporized ethanol and N_2_ as feedstock. Therefore, a direct and fair comparison of the result in this work and those from the literature is difficult. Table S2 in Supporting Information includes the relevant studies with bio-ethanol as feedstock [19,20,27,28,29,41,48,50,58,59]. As can be seen, the HZ-6NH catalyst exhibited similar or even better propene selectivity and catalyst stability under similar reaction conditions than many of the previously reported HZSM-5 catalysts. Especially, for a clear comparison of the catalytic performance between the HF-modified catalyst [28] (ZSM-5-6HF, from our previous work) and the NH_4_F-HF-modified catalyst (HZ-6NH, from this study) in the ETP reaction under the same reaction conditions, Table 4 lists the relevant parameters of different samples.

As shown in Table 4, the catalysts before and after modification showed similar maximum propylene selectivity. In terms of working life, compared to HZ, the working life of ZSM-5-6HF and HZ-6NH was prolonged to 95 h and 105 h, respectively, indicating that HZ-6NH showed an advantage in catalytic stability. The extended working life of both ZSM-5-6HF and HZ-6NH was attributed to the weakening of surface acidity, as the weakened acidity of the catalyst suppressed aromatic formation, thereby reducing coke formation. However, HZ-6NH outperformed with a longer working life, extending from 95 h for ZSM-5-6HF to 105 h. This was due to the larger mesopore volume and average pore diameter of HZ-6NH (Table 4), which facilitated the rapid diffusion of products out of the catalyst, preventing secondary reactions of aromatics, thereby reducing coke formation. This was also evidenced by HZ-6NH’s slower average coke deposition rate (Table 4). Therefore, HZ-6NH exhibited superior catalytic stability due to the improvement in diffusion performance from its pore structure.

## 5. Conclusions

The introduction of NH_4_F into the HF aqueous solution adjusted the ionization equilibrium, causing the NH_4_F-HF mixed solution to remove silicon and aluminum framework from the zeolites simultaneously, resulting in the modified catalysts having a Si/Al molar ratio similar to that of HZ. The NH_4_F-HF modification significantly altered the pore structure of the zeolite, leading to a significant increase in external specific surface area and the formation of additional mesopores. Compared to HZ, the surface acidity of the modified catalysts significantly weakened. The modified sample HZ-6NH demonstrated the longest working life, which was not only due to the weakening of acidity that suppressed coke formation and reduced the coke deposition rate, but also to the increase in mesopores that enhanced mass transfer rates and reduced secondary reactions of higher hydrocarbons like aromatics. Additionally, the significant increase in the external specific surface area and mesopore volume of HZ-6NH improved its coke tolerance, allowing it to accommodate more coke, which contributed to its superior catalytic stability. The NH_4_F-HF modification had a limited effect on the improvement of propylene selectivity, but had the advantage of significantly increasing the working life of the catalyst. The generation of coke and the weakening of acidity may be the main reasons for catalyst deactivation.

## Figures and Tables

**Table 1:** Pore properties, relative crystallinity, and Si/Al molar ratio of different samples.

Sample	Surface Area (m^2^ g^−1^)			Pore Volume (cm^3^ g^−1^)			D_P_ *^a^* (nm)	RC *^b^* (%)	Si/Al Molar Ratio *^c^*
	Total	Micropore	External	Total	Micropore	Mesopore			
HZ	326	226	100	0.19	0.12	0.07	2.30	100	16.9
HZ-2NH	336	80	256	0.24	0.05	0.19	2.92	79	17.0
HZ-6NH	318	92	226	0.25	0.05	0.20	3.18	77	16.1
HZ-8NH	337	64	273	0.27	0.04	0.23	3.24	76	16.5

*^a^* Average pore diameter; *^b^* relative crystallinity calculated from XRD patterns; *^c^* determined by ICP-OES.

**Table 2:** Initial reaction performance of different samples.

Samples	Distribution of Main Products (%)					
	Ethylene	Propylene	Butylene	Paraffins (C_1_–C_4_)	Aromatics	Others
HZ	5.2	6.0	7.2	41.9	31.0	8.7
HZ-2NH	35.5	26.8	18.9	12.4	5.0	1.4
HZ-6NH	38.9	24.2	18.8	12.1	4.6	1.4
HZ-8NH	40.5	25.6	17.5	11.1	4.4	0.9

**Table 3:** Pore properties of fresh and spent samples.

Sample	Surface Area (m^2^ g^−1^)			Pore Volume (cm^3^ g^−1^)			D_P_ *^a^* (nm)	RC *^b^* (%)	Si/Al Molar Ratio *^c^*
	Total	Micropore	External	Total	Micropore	Mesopore			
HZ	326	226	100	0.19	0.12	0.07	2.30	100	16.9
HZ-S	243	193	50	0.13	0.10	0.03	2.22	–	–
HZ-6NH	318	92	226	0.25	0.05	0.20	3.18	77	16.1
HZ-6NH-S	305	71	234	0.21	0.04	0.17	2.76	–	–

*^a^* Average pore diameter; *^b^* relative crystallinity calculated from XRD patterns; *^c^* determined by ICP-OES.

**Table 4:** Related parameters of different samples.

Sample	Mesopore Volume (cm^3^ g^−1^)	Average Pore Diameter (nm)	Average Coke Deposition Rate (g (g_cat_·h)^−1^)	Maximum Propylene Selectivity (%)	Working Life (h)
HZ	0.07	2.30	1.4 × 10^−3^	24.1	36.8
ZSM-5-6HF [28]	0.14	2.30	2.9 × 10^−4^	24.0	95.0
HZ-6NH	0.20	3.18	2.3 × 10^−4^	24.2	105.0

Reaction conditions: Ethanol concentration = 90 vol%, T = 500 °C, atmospheric pressure, WHSV = 10 h^−1^.

## Data Availability

The original contributions presented in the study are included in the article and supplementary material, further inquiries can be directed to the corresponding authors.

## Supplementary Materials

The following supporting information can be downloaded at: https://www.mdpi.com/article/10.3390/nano14191558/s1, Figure S1: DTG curves of different spent samples; Table S1: The acidity data of different samples; Table S2: Comparison of performance of different ZSM–5 zeolite catalysts.
