# Supplementary material for: Fluoride-Treated Nano-HZSM-5 Zeolite as a Highly Stable Catalyst for the Conversion of Bioethanol to Propylene
Source: Nanomaterials (Basel). 2024 Sep 26;14(19):1558. doi: 10.3390/nano14191558 (PMC11478267; doi:10.3390/nano14191558)
Supplement: Supplementary file 1 [file nanomaterials-14-01558-s001.zip › nanomaterials-3201616-supplementary.pdf]

nanomaterials

MDPI

Supplementary Materials

Fluoride-treated nano-HZSM-5 zeolite as a highly stable catalyst for the conversion of bioethanol to propylene

Jian Zhou †, Ni Zhang †, Tao Meng, Qiangsheng Guo, Zhaoteng Xue * and Dongsen Mao *

School of Chemical and Environmental Engineering, Shanghai Institute of Technology, Shanghai 201418, China; 216061106@mail.sit.edu.cn (J.Z); 146061314@mail.sit.edu.cn (N.Z); mengtao@sit.edu.cn (T.M); guo-qsh@sit.edu.cn (Q.G)

* Correspondence: ztxue@sit.edu.cn (Z.X); dsmao@sit.edu.cn (D.M)

† These authors contributed equally to this work.

Table S1. The acidity data of different samples.

Sample	Weak acid peak area (a.u.)	Strong acid peak area (a.u.)	Total acid peak area (a.u.)
HZ	146	28	174
HZ-2NH	44	9	53
HZ-6NH	45	20	65
HZ-8NH	46	25	71

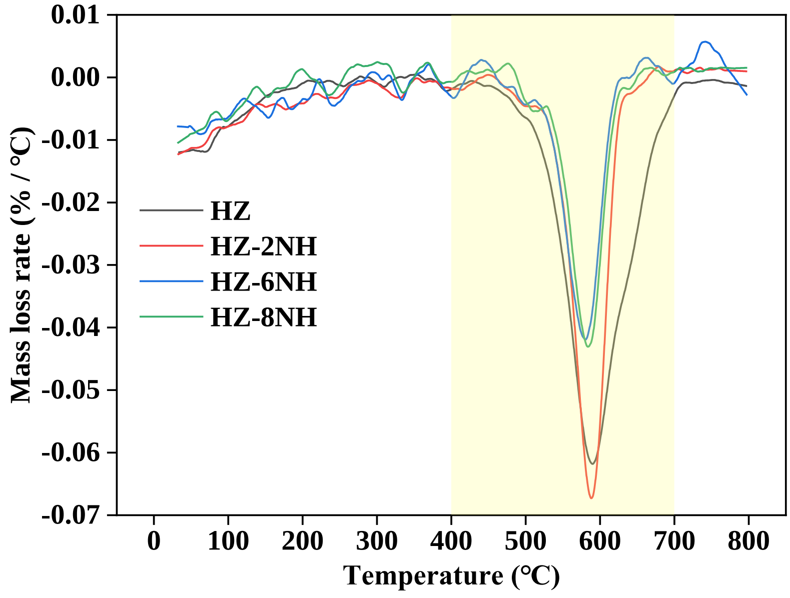

Figure S1. DTG curves of different spent samples

Table S2. Comparison of performance of different ZSM-5 zeolite catalysts.

Catalyst properties or modification methods	Process conditions	Catalytic performance*	Ref.
HF + NH_4F modification with a Si/Al molar ratio = 16.9	T = 500 °C, WHSV = 10 h^{-1} φ(Ethanol) = 90%	X = 100%, S = 24.2% L = 105 h	This work
HF modification with a Si/Al molar ratio = 16.9	T = 500 °C, WHSV = 10 h^{-1} φ(Ethanol) = 90%	X = 100%, S = 24% L =95 h	[28]
NaOH + H_3PO_4 modification with a Si/Al molar ratio = 69	T = 500 °C, WHSV = 3.3 h^{-1} φ(Ethanol) = 90%	X = 100%, S = 27% L = 100 h	[41]

Si/Al molar ratio = 50 average particle size = 100 nm	T = 500 °C, WHSV = 1.58 h^{-1} φ(Ethanol) > 99.5%	X = 100%, S = 22.4% L = 96 h	[50]
NH_4F modification with a Si/Al molar ratio = 19	T = 500 °C, WHSV = 10 h^{-1} φ(Ethanol) = 90%	X = 100%, S = 24.9% L = 214 h	[27]
Si/Al molar ratio = 70 average particle size = 100 nm	T = 500 °C, WHSV = 1.58 h^{-1} φ(Ethanol) > 99.5%	X = 100%, S = 24% L = 57 h	[29]
NaOH + $Mg(CH_3COO)_2$ modification with a Si/Al molar ratio = 38	T = 500 °C, WHSV = 3.3 h^{-1} φ(Ethanol) = 90%	X = 100%, S = 26.6% L = 62.8 h	[20]
NaOH modification with a Si/Al ratio = 30	T = 400 °C, WHSV = 0.136 h^{-1}, φ(Ethanol) = 95% TOS = 22 h	X = 100% S = 30% (propene + butene)	[19]
$Ni(NO_3)_2$ modification with a Si/Al ratio = 30	T = 500 °C, WHSV = 0.3 h^{-1} φ(Ethanol) = 75%	X = 100%, S = 19%	[48]
$Ni(NO_3)_2·6H_2O$ modification with a Si/Al ratio = 30	T = 500 °C WHSV = 0.136 h^{-1} φ(Ethanol) = 95%	X = 100% S = ~41% (propene + butene)	[58]
NaOH modification with a Si/Al ratio = 30	T = 400 °C, WHSV = 0.235 h^{-1}, φ(Ethanol) = 50% TOS = 14 h	X = 100% S = ~35% (propene + butene)	[59]

* X, S, L: conversion, selectivity, and working life, respectively.
